# Examination Questions before the State Medical Examining Board

**Published:** 1899-05

**Authors:** 


### EXAMINATION QUESTIONS BEFORE THE STATE MEDICAL EXAMINING BOARD.

WE append a list of the examination questions recently given to candidates by the Medical Examining Board. The first are those submitted at the examination held in Augusta, and the second are those given in Atlanta. We are sorry not to be able to give a tabulated list of the colleges who sent representatives, and also the marks, but the Secretary, Dr. Anthony, has written us that it was impossible to get them up in shape for publication. There were about forty who stood the examination in Augusta and about fifty in Atlanta. Out of the total number there were about four failures.

We give the Questions:—

*Regular Board of Examiners. Augusta, Ga., April 3, 1899.*

MATERIA MEDICA AND THERAPEUTICS.—BY DR. W. A. O'DANIEL.

1. By what should you be governed in the administration of drugs?

2. How are drugs classified? Give an example of each.

3. What is cocain? How would you treat cocain poisoning?

4. What are the physiological effects of bromide potassium?

5. Tell what you know of the origin, preparations, and physiological effects of nux vomica?

6. Give doses of following drugs: Acid. arseniosum, hydrarg. chlor. corro., zinci phosphidum.

CHEMISTRY.—BY DR. E. R. ANTHONY.

1. Define symbol, formula, element.

2. What is a glucosid? Name three or four used in medicine.

3. Name and give the formulas of two important salts used in medicine.

4. Give a good test for bile in the urine.

5. What is the composition of biliary calculi?

PHYSIOLOGY.—BY DR. E. R. ANTHONY.

1. Mention four or five varieties of connective tissue and tell where found.

2. Define excretion. Where is the seat of urea formation and how is it eliminated?

3. What forces cause the circulation of the blood through the arteries? the veins? the capillaries?

4. What changes take place in the blood in respiration?

5. What effect has section of the antero-lateral columns?

SURGERY.—BY DR. F. M. RIDLEY.

1. (a) Describe amputation of the hip joint by the Wyeth method. (b) How should a compound fracture of the leg be treated? (c) Describe Colles's fracture, Pott's fracture, and give approved treatment of each.

2. (a) What is intussusception? (b) Volvulus. Give rational treatment. (c) Describe an approved method of internal anastimosis. (d) Give treatment of gunshot wounds of abdomen.

3. (a) Describe carcinoma, sarcoma, epithelioma. Describe operation for removal of breast. (b) What are the essential features aimed at in Halstead's operation?

4. What is hydrocele? What is hernia. Give differential diagnosis. How should a hydrocele be treated? Describe operation for hydrocele. What is varicocele? Describe operation for its cure.

5. What is inflammation? Give pathology of erysipelas, and give treatment. Describe malignant pustule and give treatment.

ANATOMY.—BY DR. F. M. RIDLEY.

1. (a) Describe situation and division of temporal bone. (b) Give articulations. (c) Give articulations of ethmoid bone. (d) Name bones of carpus and give articulations.

2. (a) What characteristics are common to vertebræ? (b) Name divisions of vertebral column.

3. (a) There are nine peculiar vertebræ—what are they? (b) Describe by diagram the triangles of the neck.

4. (a) Name the cranial nerves. (b) Name those which have their origin wholly or in part from the floor of the fourth ventricle. (c) Name ductless glands. (d) Enumerate the ligaments of the uterus.

5. (a) Describe peritoneum. (b) Is it always a shut sac? (c) What and where is the foramen of Winslow? Name and illustrate regions of abdomen. What is contained in each?

GYNECOLOGY.—BY DR. JAMES W. BAILEY.

1. What function of the uterus is of most interest to the gynecologist.

2. What is menstruation? How should it recur? What is the average time for its recurrence?

3. Describe briefly the operation of myomectomy.

4. What is a parovarian cyst? Give location and diagnosis.

5. What is amenorrhea? Cause, symptoms, and treatment.

OBSTETRICS.—BY DR. JAMES W. BAILEY.

1. Describe briefly the characteristic signs and symptoms of pregnancy.

2. What treatment would you pursue in inevitable abortion?

3. Give different methods of treatment in postpartum hemorrhage.

4. In placenta previa, what signs and symptoms demand attention? What mode of treatment would you adopt?

5. How would you differentiate between vertex and breech presentation?

PRACTICE.—BY DR. A. A. SMITH.

1. Give prominent symptoms and appropriate treatment for pericarditis.

2. Give differential diagnostic symptoms in spasmodic and membranous croup.

3. What does the term hemiplegia denote?

4. Give differential diagnostic symptoms in diarrhea and dysentery.

5. Give symptoms in acute pleuritis, and appropriate treatment for same.

6. Give a few prominent symptoms of typhoid fever.

7. What do you understand by the term articular rheumatism?

8. Give symptoms of acute tonsillitis and treatment for same.

*Regular Board of Examiners, Atlanta, Ga., April 4, 1899.*

MATERIA MEDICA AND THERAPEUTICS.—BY DR. W. A. O'DANIEL.

1. What is glonoin? Give dose and indications for its use.

2. What effect has digitalis upon the heart?

3. Give dose and therapeutic indications for acetanilid?

4. Tell me all you know about ferrum?

5. When are emetics indicated and when contraindicated? Give dose of some local emetic.

6. Give the dose of following drugs: apomorphine, atropine sulphas, hyoscine.

CHEMISTRY.—BY DR. E. R. ANTHONY.

1. Define valence, salt, base.

2. What is an alkaloid? Name three or four used in medicine.

3. Name and give the formulas of two important acids used in medicine.

4. Give a good test for pus in the urine.

5. Give the composition of two or three of the most common urinary calculi.

PHYSIOLOGY.—BY DR. E. R. ANTHONY.

1. Mention four or five varieties of epithelia and tell where found.

2. Define secretion. Mention three secretions and give the function of each.

3. Give the structural differences between arteries, veins and capillaries.

4. What changes take place in the air in respiration?

5. What effect has section of a lateral half of the spinal cord?

SURGERY.—BY DR. F. M. RIDLEY.

1. What is an abscess? What is an aneurism? Give differential diagnosis. What is septicemia, sapremia, pyemia? What do we understand by ptomaines and leucomaines?

2. What is fracture? Differential diagnosis between fracture and dislocation? Name varieties of fracture. What are the cardinal principles in the treatment of fractures? Give approved method for treatment fractured patella.

3. What is hernia? What is an inguinal hernia? Name from without inward the coverings of an oblique inguinal hernia? Describe an approved operation for the radical cure.

4. What is erysipelas? Give pathology and treatment. What is Pott's disease? Give etiology, diagnosis and treatment.

5. What is intussusception? Describe an approved method for intestinal anastimosis. Give differential diagnosis between chancre and chancroid, and treatment of each.

ANATOMY.—BY DR. F. M. RIDLEY.

1. Name bones of face. Give articulations of spheroid bone. What is a sesamoid bone. Name them. Name bones of leg.

2. Describe the abdominal aorta. Name its branches. Describe briefly the common iliac artery.

3. How is the nervous system divided? Of What does the cerebrospinal system consist?

4. Describe the sympathetic nerve.

5. Name the ganglia of the sympathetic in the cranium and its vicinity. Name and illustrate by diagram the regions of the abdomen. What is contained in each?

GYNECOLOGY.—BY DR. JAS. W. BAILEY.

1. Describe vesicouterine fistula; give cause and treatment.

2. What is the difference between corporeal and cervical endometritis?

3. What is hysterectomy?

4. Define anteflexion of the uterus. How many forms? Describe each.

5. What is rectocele?

OBSTETRICS.—BY DR. JAS. W. BAILEY.

1. How many bones in the pelvis? Name them.

2. How is the pelvis divided? Give divisions.

3. What are the organs of generation in the female?

5. How many stages of labor? Describe each.

5. Give mode in which expulsion of the child is effected.

PRACTICE.—BY DR. A. A. SMITH.

1. Give symptoms and appropriate treatment for cerebrospinal meningitis.

2. Give technical name for lockjaw, and treatment for same.

3. What is endocarditis?

4. What form of paralysis is paraplegia?

5. If in examining a specimen of urine you find it to contain sugar and the specific gravity 1030 or above, what would be your diagnosis?

6. Give symptoms of acute gastritis, and treatment for same.

7. Give prominent symptoms and treatment for cholera infantum.

8. Give symptoms and appropriate treatment for acute peritonitis.

9. What would you expect to find by examining the throat in diphtheria?

